# Complete Genome Sequence of a Rare Pigment-Producing Strain of Acinetobacter johnsonii, Isolated from the Bile of a Patient in Hangzhou, China

**DOI:** 10.1128/mra.00025-22

**Published:** 2022-04-04

**Authors:** Xinli Mu, Haiyang Liu, Yue Yao, Feng Zhao, Youhong Fang, Yunsong Yu, Xiaoting Hua

**Affiliations:** a Department of Infectious Diseases, Sir Run Run Shaw Hospital, Zhejiang University School of Medicine, Hangzhou, Zhejiang, China; b Key Laboratory of Microbial Technology and Bioinformatics of Zhejiang Province, Hangzhou, Zhejiang, China; c Regional Medical Center for National Institute of Respiratory Diseases, Sir Run Run Shaw Hospital, Zhejiang University School of Medicine, Hangzhou, Zhejiang, China; d Department of Clinical Laboratory, Sir Run Run Shaw Hospital, Zhejiang University School of Medicine, Hangzhou, Zhejiang, China; e Department of Gastroenterology, The Children’s Hospital, Zhejiang University School of Medicine, National Clinical Research Center for Child Health, Hangzhou, Zhejiang, China; University of Arizona

## Abstract

Here, we report the complete genome sequence of a rare pigment-producing strain of Acinetobacter johnsonii. The genome consists of a 3,360,823-bp circular chromosome (G+C content, 41.56%) and an 8,887-bp plasmid (G+C content, 33.71%). It possesses 3,038 coding gene sequences, 19 rRNA genes, 87 tRNA genes, and 4 noncoding RNA (ncRNA) genes.

## ANNOUNCEMENT

Acinetobacter johnsonii is an opportunistic human pathogen found in natural and nosocomial environments, such as agricultural soil (1, 2). Two draft genome sequences were previously reported, from a creosote-contaminated aquifer and a frost-plant tissue sample (3, 4), but there have been no reports describing pigment production in this species. Moreover, little is known about whether this species causes infections in patients in healthcare settings, especially for the strain with the special pigment phenotype. Here, we report the complete genome sequence of a rare pigment-producing strain of *A. johnsonii*, collected on 14 March 2021 from the bile of a patient in Hangzhou, China.

**FIG 1:**
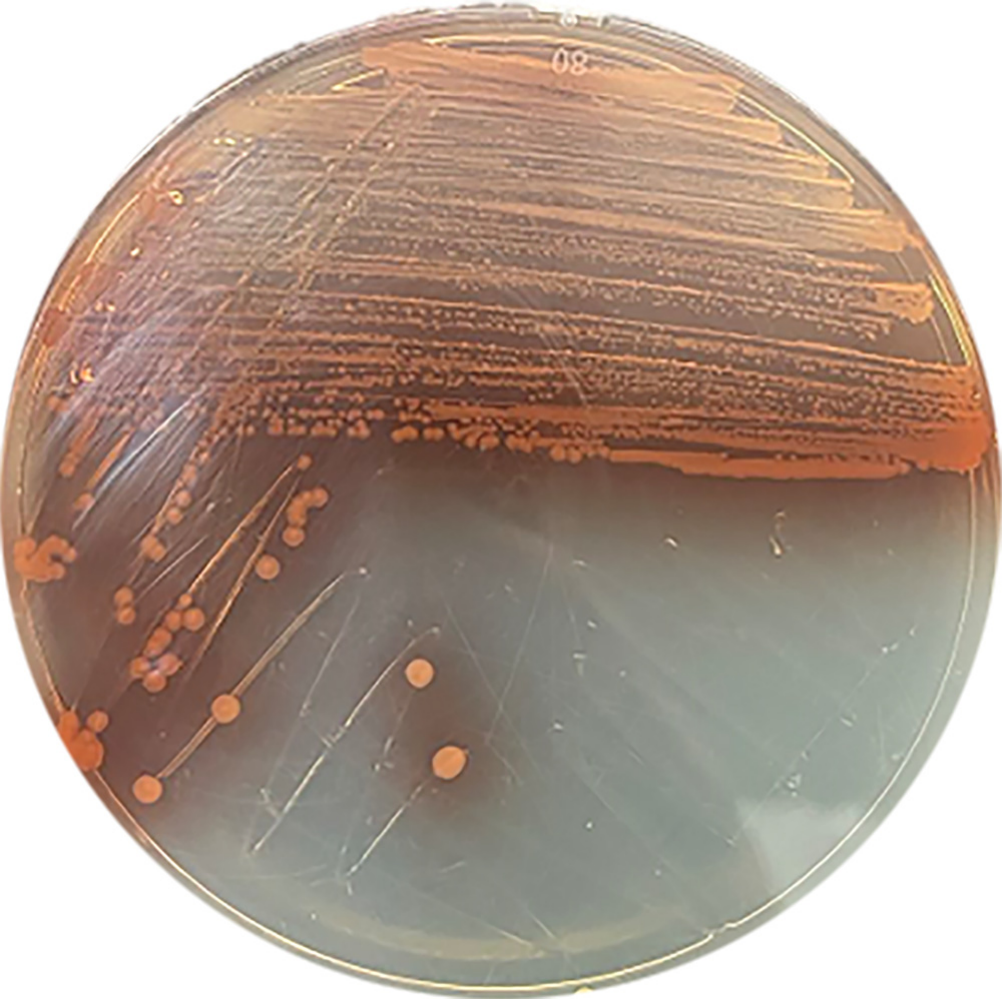
Color and morphology of a rare pigment-producing strain of *A. johnsonii* DJ-Red. Bacteria were cultured on a Mueller-Hinton (MH) agar plate for 2 days at 37°C under static incubation. A reddish-brown pigment could be seen after culture.

*A. johnsonii* DJ-Red was isolated during routine diagnostic analysis. (Consent for sampling was obtained from the patient.) The strain was cultured on a Mueller-Hinton agar (MHA) plate (Oxoid, Hampshire, UK) and incubated at 37°C for 24 h. Then, a single colony was picked and grown in MH broth (Oxoid) with shaking at 200 rpm/min at 37°C for 24 h. Considering its special pigment phenotype (Fig. 1), genomic DNA was extracted using the Qiagen minikit (Germany) and the Gentra Puregene yeast/bacteria kit (Qiagen) for further Illumina and Nanopore sequencing, respectively. DNA library preparation for Illumina sequencing was performed using the NEXTFLEX rapid DNA-Seq kit, and sequencing was conducted on the HiSeq X Ten platform (Illumina Inc., San Diego, CA, USA). Trimmomatic v0.30 was used to trim the Illumina reads (5). Libraries for Nanopore sequencing were prepared using the SQK-LSK109 ligation sequencing kit in conjunction with the PCR-free Oxford Nanopore Technologies (ONT) native barcode expansion kit (EXP-NBD104), and sequencing was performed without the optional shearing steps to select for long reads. The individual libraries were quantitated using a Qubit 3.0 fluorometer (Invitrogen, Carlsbad, CA, USA). Finally, the library was loaded onto an R9.4.1 flow cell and sequenced on the MinION platform (Oxford Nanopore Technologies, UK). Hybrid assembly of the filtered Nanopore and Illumina reads was performed using Raven v1.1.10, and error correction was conducted using Pilon v1.24 (6, 7). Genome annotation was performed by the National Center for Biotechnology Information (NCBI) Prokaryotic Genome Annotation Pipeline (PGAP) (http://www.ncbi.nlm.nih.gov/genome/annotation_prok/) (8). Antimicrobial resistance genes and virulence genes were identified using ABRicate v0.8.13 (https://github.com/tseemann/abricate) with the ResFinder database and Bacterial Virulence Factor Database (VFDB) (9, 10). Insertion sequences (ISs) were identified using ISfinder (11). PHAge Search Tool (PHAST) was utilized for the prediction of bacteriophages (12). Default parameters were used for all software unless otherwise specified.

The depth of the Illumina sequencing averaged 308×; it yielded a total of 2,969,045 150-bp paired-end reads. The Nanopore sequencing coverage averaged 545×, and a total of 85,967 reads were obtained, with an average length of 8,240 bp and an *N*_50_ value of 13,540 bp. The genome assembly data indicated a 3,360,823-bp circular chromosome with a G+C content of 41.56% and one 8,887-bp plasmid (pDJ-Red) with a G+C content of 33.71% (Table 1). Plasmid circularization was conducted using Raven. Furthermore, the replication initiation protein gene *repE* was identified on pDJ-Red. The 16S rRNA sequence of *A. johnsonii* DJ-Red was 99.65% identical to that of *A. johnsonii* ATCC 17909^T^ (GenBank accession number Z93440.1). Using the online average nucleotide identity (ANI) tool (http://enve-omics.ce.gatech.edu/ani/index), analysis showed a 96.35% two-way ANI between *A. johnsonii* DJ-Red and *A. johnsonii* LXL_C1 (CP031011.1), revealing that *A. johnsonii* DJ-Red belongs to this species. Genome annotation predicted 3,038 protein-coding genes, 19 rRNA genes (7 5S rRNAs, 6 16S rRNAs, and 6 23S rRNAs), 87 tRNA genes, 4 ncRNA genes, and 119 pseudogenes (Table 1). Moreover, *bla*_OXA-662_ (in the OXA-211 family), an intrinsic antibiotic resistance gene, was identified. The genes *lpxC* and *bfmR*, found on the chromosome of *A. johnsonii* DJ-Red, encode two virulence factors involving lipopolysaccharide (LPS) and a two-component system, respectively. The genome also contains four incomplete prophage regions, with scores of 60, 40, 60, and 50. Importantly, several ISs, including the IS*5*, IS*3*, and IS*30* families, were discovered. The presence of prophage regions and ISs may accelerate the evolution of mobile genetic elements of *A. johnsonii* strains with this special phenotype.

**TABLE 1:** Genome features and annotation statistics of *A. johnsonii* strain DJ-Red

Genome feature	Data for:	
	Chromosome	Plasmid (pDJ-Red)
Genome size (bp)	3,360,823	8,887
G+C content (%)	41.56	33.71
No. of rRNAs	19	0
No. of tRNAs	87	0
No. of ncRNAs^*a*^	4	0
No. of coding sequences	3,027	11
GenBank accession no.	CP090180	CP090181

a ncRNAs, noncoding RNAs.

The complete genome sequence of *A. johnsonii* DJ-Red will promote the understanding of nosocomial infections caused by this species and offer new insight into treatment.

### Data availability.

The complete sequences of the chromosome and plasmid from *A. johnsonii* DJ-Red have been deposited at GenBank in the nucleotide database under accession numbers CP090180 and CP090181, respectively. The raw sequence data have been deposited in the Sequence Read Archive (SRA) under accession number SRR17615989.
